# Antibodies against oxidation-specific epitopes and risk of acute myocardial infarction

**DOI:** 10.1016/j.jlr.2023.100412

**Published:** 2023-07-16

**Authors:** Johan Frostegård

**Affiliations:** Unit of Immunology and Chronic Disease, IMM, Karolinska Institutet, Stockholm, Sweden

**Figure:**
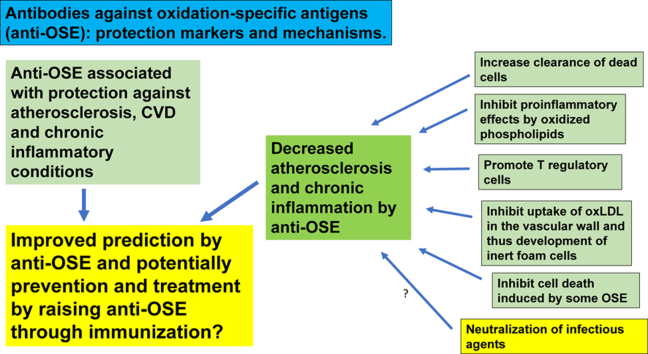

Atherosclerosis and its major consequence, myocardial infarctions, represent a major cause of morbidity and mortality. Atherosclerosis is a chronic inflammatory process in the intima of most large- and middle-sized arteries (1). Typical of atherosclerosis is the accumulation of oxidized lipid moieties, mainly from oxidized low density lipoprotein (OxLDL), in the intima of many arteries. According to the LDL-oxidation hypothesis, OxLDL is involved in the pathogenesis of atherosclerosis and CVD. This was proposed in the 80s, as an extension and development of the concept of LDL being a risk factor (2). Another major feature of atherosclerosis is the accumulation of dead cells in lesions. Atherosclerosis could therefore be described as a problem of defective clearance since the body normally has strong defense and clearance systems for these types of compounds.

It has been known for long time that atherosclerosis is an inflammatory process, where immune competent cells accumulate, initially in the intima of arteries. Cell types believed to be of major importance in atherosclerotic plaques include activated dendritic cells, monocytes/macrophages, and T-cells producing mainly proinflammatory cytokines. Also, endothelial cells and smooth muscle cells play a role (3).

One driver of the chronic low-grade inflammation in atherosclerosis could be OxLDL, which is known to be both immunogenic and proinflammatory under certain circumstances and also toxic for cells (4), but there could be others, including necrotic cells and even infectious agents (1).

During LDL oxidation, several interesting and potentially atherogenic compounds are generated, including lipid-related ones malondialdehyde (MDA), phosphorylcholine or phosphocholine (PC), and apoB100-related ones (apoB being the protein in LDL). Oxidation can occur by different nonmutually exclusive mechanisms, including chemical oxidation and enzymatic modification (1).

Antibodies against oxLDL (anti-OxLDL) have been known for a long time, but their role in atherosclerosis is complex. In the early 90s, reports mostly indicated anti-OxLDL as a risk marker for atherosclerosis and its complications, and anti-OxLDL was even demonstrated to be cross-reacting with thrombogenic antiphospholipid antibodies (5). After that, an association between antibodies and protection in relation to atherosclerosis or CVD was reported for the first time, for anti-OxLDL (6).

One way of dealing with the complexity of OxLDL and anti-OxLDL is to study antibodies against components of OxLDL or related compounds, which is the topic of the paper entitled “High immunoglobulin M levels to oxidation-specific epitopes are associated with lower risk of acute myocardial infarction” in this issue of the *Journal of Lipid Research*, which focuses on oxidation-specific epitopes (OSEs). MDA is studied both as a part of MDA-OxLDL and as a peptide mimotope with similar antigenic properties. PC is by itself only a hapten but becomes immunogenic when exposed as in oxLDL or bound to a carrier (here bovine serum albumin. Another component of LDL, apoB100, is also studied as an antigen herein. All four were lower among patients with acute myocardial infarction (AMI) when controlled for other risk factors, and associations were stronger when high and low percentiles were compared.

The present study was carried out in order to investigate the role of these antibodies in myocardial infarction, and also to simultaneously study these antigens in the same setting, so they could be compared. The study, thus far the largest on this topic, consists of a cohort of 4,559 patients with AMI and 4,617 age- and sex-matched controls without AMI were compared. IgM levels were measured within 24 h of first AMI. By the use of C-statistics, it was demonstrated that IgM against OSE significantly adds strength to established risk factors in determining risk of AMI. Importantly, IgM against OSE were specific as a protection marker since total IgM did not give such an association.

Both environmental and genetic factors contribute to the continuous production of IgM anti-OSE but details of this need to be more clarified. These antibodies are likely to be pivotal both in protection against infections and in cellular/organ homeostasis, including clearance of debris and dead cells. This type of antibodies have previously been reported to be associated with protection not only against atherosclerosis and its complications but also against other chronic inflammatory conditions and autoimmune diseases (1).

One limitation of this study is that it only focuses on IgM. Even though IgM against OSE represents 20–30% of the circulating IgM pool in the absence of acute infections, other subclasses and isotypes could be of interest. IgG1 anti-PC is similar to IgM is in association with protection with CVD, while IgG2 is less so (7). Further studies on these are clearly needed. Another limitation is that the study is not prospective, and large studies of incident cases of CVD and the role of these types of antibodies are needed to further establish anti-OSE antibodies as determinants of risk.

Animal experiments support a protective role of this type of antibodies. For example, immunization with MDA-modified LDL ameliorated atherosclerosis development in both rabbits (8) and mice (9) and immunization with PC decreases atherosclerosis development using active (10) or passive (11) immunization. There are also several underlying mechanisms which could explain how these antibodies could be protective, with the three most important appearing to be an anti-inflammatory effect, inhibition of OxLDL uptake in macrophages in the artery wall leading to foam cell formation, and increased clearance of dead cells (1).

There are interesting similarities, but also differences between these OSEs. All are danger-associated molecular patterns (DAMPs), signaling to different defense systems in the body that the compound to which they are exposed needs to be neutralized and/or removed. PC, though, is not only a DAMP but also a pathogen-associated molecular pattern (PAMP). To the best of my knowledge, neither MDA or apoB100 play an important role if any, as PAMPs. PC is exposed as a PAMP on many bacteria, including the polysaccharide capsule of Streptococcus pneumoniae. PC is also an important antigen in other microorganisms such as nematodes and parasites.

Heritability is relatively high for antibodies against OSE in general (67%) (12), while lower for anti-PC (we reported 40%) (13) which may reflect a substantial influence by pathogens and/or the microbiome on anti-PC levels. This makes PC a potentially even more interesting vaccine candidate to increase protection against atherosclerosis and its complications since we are already immunized “naturally” by such external and microbial antigens.

An example of natural immunization is observed in brown bears (Ursus arctos), which during hibernation show no signs of atherosclerosis in spite of uremia, immobilization, and very high cholesterol levels, all strong risk factors. They have much higher anti-PC levels during the winter than in summer. This is not the case for anti-MDA. As MDA is only a DAMP, not a PAMP, this increase in anti-PC levels may be caused by the bears’ behavior, exposure to PC-bearing antigens, and consumption pattern during the preparation for hibernation, including being exposed to PAMPs (14) Similarly, IgM anti-PC levels are much higher among individuals from Papua New Guinea, living a life as hunters, gatherers, and horticulturalists and with little signs of CVD as compared to age- and sex-matched Swedes (15).

In line with this reasoning, humans are born with substantial amounts of IgM anti-MDA, while anti-PC is almost nondetectable at birth and thus requires contact with the external world to develop (16). Furthermore IgM anti-PC is not germ line encoded with dominating clone as TI5 in mice, instead humans mount somatically mutated antibodies against PC using a broad variety of Ig genes (17).

Interestingly, women have higher levels of these antibodies than men in all studies I am aware of. This is interesting given that women get CVD some years later in life, than men, which would support the notion of a protective role. The cause of womens’ higher levels of this type of antibodies is interesting per se and could have different causes, which are not well described.

Taken together, this interesting study adds strength to the notion that increasing antibody levels against OSE, through active and even passive immunization, could ameliorate atherosclerosis and decrease the risk of complications of atherosclerosis like myocardial infarction as in the present paper but also stroke and other chronic inflammatory conditions.

## Conflict of interest

The author declares that they have no conflicts of interest with the contents of this article.
